# Working Diagnoses: A Pilot Study

**DOI:** 10.1192/bjo.2025.10404

**Published:** 2025-06-20

**Authors:** Zaim Mohdesham, Ben di Mambro

**Affiliations:** 1 Derbyshire Healthcare NHS Foundation Trust, Derby, United Kingdom; 2 Nottinghamshire Healthcare NHS Foundation Trust, Nottingham, United Kingdom

## Abstract

**Aims:** Mental Health and Neurodevelopment Resource Group (MHNRG) are planned to replace Mental Health Clustering. However, they are broad diagnostic groupings which will potentially have limited benefit in relation to evaluating outcomes, health inequalities, pathways, and interventions. In addition to mandatory completion of MHNRG, local services have the option to collect additional categorical data which led to the introduction of Working Diagnoses.

This is a pilot study to trial Working Diagnoses to test its functionality and feasibility.

**Methods:** The aim of the Working Diagnoses is to create an accessible form on the electronic patient record allowing assessors to select a list of up to four working diagnoses via a drop-down menu. Following consultation with clinicians from differing psychiatric specialities, a list of 53 separate working diagnoses were agreed upon which were individually mapped to their respective ICD–11 diagnostic codes and Systematized Nomenclature of Medicine Clinical Terms (SNOMED CT) to make it future proof.

The pilot was conducted within the local Crisis Resolution and Home Treatment and Primary Care Mental Health teams. A live and secure Microsoft Excel document with a list of the working diagnoses through a drop-down menu was created. Assessors consisting of both doctors of various grades and psychiatric nurses within the teams were briefed on the aims and objectives of the pilot study.

At this stage, it is not intended for the diagnostic data to flow into the Mental Health Services Data Set (MHSDS).

**Results:** 127 patients referred to the teams between November to December 2023 were included in the pilot study and allocated their working diagnosis; 66 received one diagnosis, 52 received two and 9 received three diagnoses and none received four.

All presentations were able to be satisfactorily described by the Working Diagnoses options. The general feedback from assessors who participated in the study reported that it was simple and easy to use despite having limited formal training.

**Conclusion:** We believe that mental health services require granular details of a person’s presentation if we are to effectively commission, transform and manage our services. Though other options could be utilised, implementing a limited categorical diagnostic list appears to be an acceptable, effective, and efficient method of gathering the information that has been missing in mental health services locally.

The next steps will be to trial this to other services within the wider trust.

